# Size-selective microfluidics delineate the effects of combinatorial immunotherapy on T-cell response dynamics at the single-cell level

**DOI:** 10.1038/s41378-024-00769-3

**Published:** 2024-11-26

**Authors:** Ayan Chatterjee, Aniket Bandyopadhyay, Tapas Kumar Maiti, Tarun Kanti Bhattacharyya

**Affiliations:** 1 Advanced Technology Development Centre, Indian Institute of Technology Kharagpur, Kharagpur, India; 2 Institute of Cell Dynamics and Imaging, University of Münster, Münster, Germany; 3 Department of Biotechnology, Indian Institute of Technology Kharagpur, Kharagpur, India; 4 Department of Electronics and Electrical Communication Engineering, Indian Institute of Technology Kharagpur, Kharagpur, India

**Keywords:** Electrical and electronic engineering, Chemistry

## Abstract

Cellular communication at the single-cell level holds immense potential for uncovering response heterogeneity in immune cell behaviors. However, because of significant size diversity among different immune cell types, controlling the pairing of cells with substantial size differences remains a formidable challenge. We developed a microfluidic platform for size-selective pairing (SSP) to pair single cells with up to a fivefold difference in size, achieving over 40% pairing efficiency. We used SSP to investigate the real-time effects of combinatorial immunotherapeutic stimulation on macrophage T-cell interactions at the single-cell level via fluorescence microscopy and microfluidic sampling. While combinatorial activation involving toll-like receptor (TLR) agonists and rapamycin (an mTOR inhibitor) has improved therapeutic efficacy in mice, its clinical success has been limited. Here, we investigated immune synaptic interactions and outcomes at the single-cell level in real time and compared them with bulk-level measurements. Our findings, after tracking and computationally analyzing the effects of sequential and spatiotemporal stimulations of primary mouse macrophages, suggest a regulatory role of rapamycin in dampening inflammatory outputs in T cells.

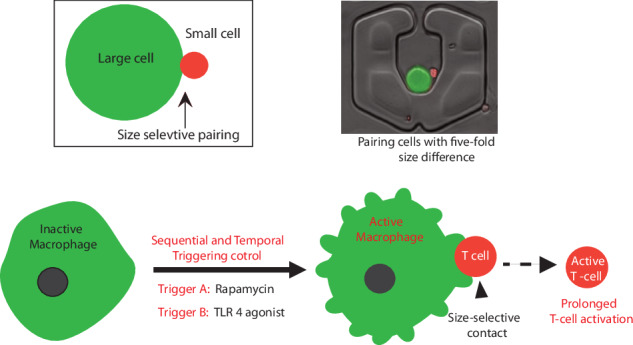

## Introduction

Interactions among diverse immune cell types within lymphatic and nonlymphatic organs present a complex landscape, often occurring among cells with significant differences in size. Traditional ex vivo studies using conventional coculture methods face challenges in maintaining continuous contact between these cells over time, rendering the tracking of short- and long-term outcomes nearly impossible. To address this issue, various microfluidic platforms have been developed to facilitate controlled interactions between immune cells with substantial size disparities.

Existing techniques involve chemical bridging via biotin-avidin chemistry^1^, electric field-driven conjugation^2^, dielectrophoretic pairing^3,4^, and magnetic dipole-mediated cell pairing^5^. While attempts have been made to use methods such as surface acoustic waves^6,7^ and optical tweezers^8^, challenges such as spatial resolution and cell damage during prolonged use persist. More recently, centrifugal force-based capture and pairing of single cells^9^ and combined hydrodynamic and recirculation flow-based capture^10^ have been reported; however, these methods cannot pair cells with significant size differences. Hydrodynamic cell pairing methods and deformability-based traps^11,12^ have been introduced to partially overcome these limitations. Shaik et al., reported for the first time a device for pairing single cells with large size differences^13^. However, the device has a complex three-layer design and requires fluid manipulations with four different inlets to control cell pairing. Additionally, the device has a low density of traps per unit area, which affects throughput.

To address this gap, we introduced a novel microfluidic size-selective pairing platform (SSP), with a single inlet and outlet system with densely distributed pairing microstructures to pair cells with a size difference of up to fivefold. To assess the efficacy of our design, we conducted a study on the impact of combinatorial immunotherapeutic stimulation on primary mouse macrophages and their interaction with OT-1 CD8+ T lymphocytes.

The aim of this study was to elucidate the effects of stimulation on ex vivo-modified antigen-presenting cells and T-cell responses, with clinical importance in the context of limited success with autologous ex vivo-modified dendritic cell vaccines after the approval of Sipuleucel T in 2010^14^. Failure at the clinical level may stem from challenges requiring extensive tracking of T-cell activities, from initial contact with antigen-presenting cells to subsequent secretory activities^15,16^.

To delve into the intricacies of combinatorial pharmacological stimulation, primary macrophages were subjected to rapamycin stimulation alongside stimulation with toll-like receptor (TLR) ligands. Notably, lipopolysaccharide (a TLR4 agonist) induces the activation of the mammalian target of rapamycin class 1 (mTORC1) pathway in human and murine macrophages as well as dendritic cells (DCs), culminating in the secretion of the anti-inflammatory cytokine IL10^17,18^ This cascade underscores the pivotal role of mTOR downstream in TLR-driven signaling pathways, dictating metabolic exigencies and fostering cell survival in dendritic cells and macrophages. Intriguingly, rapamycin elicits proinflammatory cytokine secretion, a departure from the anti-inflammatory bias typically induced by TLR agonists in these immune cells^17–19^. Strategic inhibition of mTORC1 signaling via rapamycin in response to lipopolysaccharide (LPS) prolongs the lifespan of dendritic cells while sustaining the expression of costimulatory receptors, thereby increasing the efficacy of antitumor responses in murine models^20^. Regrettably, despite promising preclinical outcomes, the clinical translation of this therapeutic strategy remains elusive. The aim of the current endeavor was to dissect and model the intricate effects of these types of antigen-presenting cell stimulation on the activation dynamics of T cells at the single-cell level, paving the way for nuanced therapeutic interventions.

Using our SSP platform, we meticulously examined the effects of temporally and sequentially regulated treatments involving the coadministration of rapamycin and LPS on primary murine macrophages. Our findings revealed that the early introduction of rapamycin robustly attenuated the anti-inflammatory bias triggered by LPS, thereby prolonging the stimulatory effects and augmenting the cytotoxic potency of OT-1 CD8+ T lymphocytes, even up to 96 h posttreatment.

## Results

### Microfluidic size-selective single-cell pairing platform

**Fig. 1:**
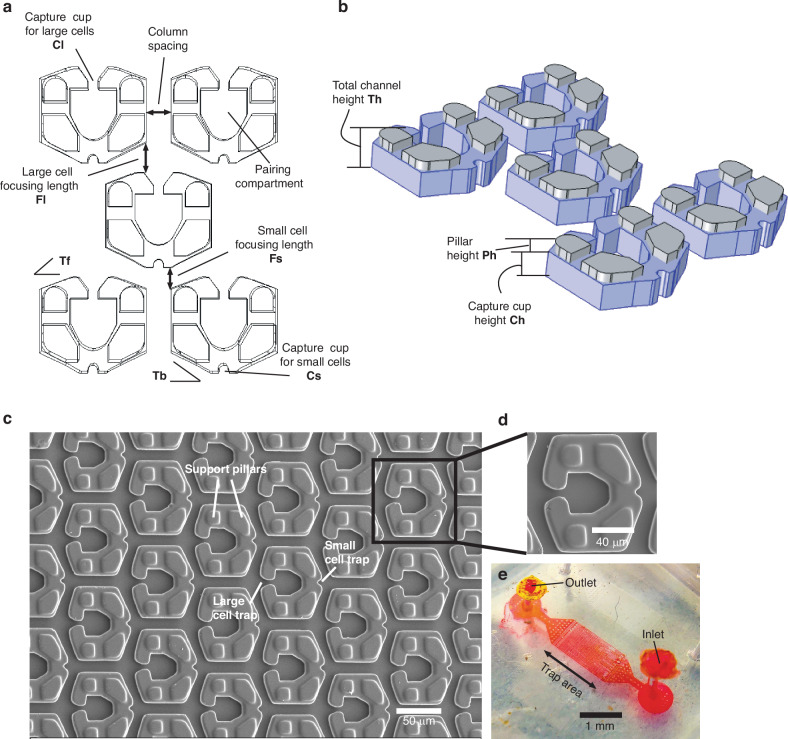
Device design. **a** Microconfinement design details. **b** Microconfinement in 3 dimensions showing the pillar height Ph and capture cup height Ch. **c** Scanning electron microscope image of the trap array. **d** Scanning electron microscope image of a single enlarged trap. **e** Image of the microfluidic device. The scale bar in (**c**) is 50 µm, that in (**d**) is 40 µm and that in (**e**) is 1 mm

Here, we report a microconfinement design for hydrodynamically capturing and pairing single cells that differ in size by up to fivefold with a single inlet and outlet configuration. As shown in Fig. 1a, each microconfinement consists of two-cell capture confinement, one for capturing large cells (Cl) and the other for capturing smaller cells (Cs) and a cell-pairing compartment (Cp). Support pillars are placed on either side of each confinement to increase fluid flow inside the capture cups. The column spacing was held constant at 1.25 times the large cell size. As shown in Fig. 1a, there are two angular tapers, one at the large-cell capture junction (Tf) and one near the small-cell capture junction (Tb). Tapering ‘Tf’ is used to define the focusing length (Fl) for larger cells, and tapering ‘Tb’ is used to define the appropriate focusing length Fs for smaller cells.

The support pillar height (Ph) and capture cup height (Ch), as shown in Fig. 1b, were varied to control fluid flow inside and around the microconfinement. The total channel height (Th) is the summation of Ph and Ch. To maximize the cell capture and pairing efficiencies, it is important to optimize the fluid flow inside the pairing compartment ‘Cp’ by varying the ratio (X = Ph/Th), as shown in Supplementary Table T1. The fabricated microconfinement array is shown in Fig. 1c, with an enlarged view of single microconfinement in Fig. 1d and an image of the complete device in Fig. 1e. There are 1000 capture traps within a 1 mm by 4.5 mm area of the device.

Owing to the introduction of two tapering angles, it is possible to achieve different focusing lengths for small and large cells. As shown in Supplementary Fig. S1a–c, the tapering angle ‘Tf’ was varied to introduce different ‘Fl’ values, whereas ‘Fs’ was kept the same by keeping the angle ‘Tb’ constant. Because we paired macrophages (average diameter 16 µm) with T cells (diameter 3 to 4 µm), the Fl varied from 20 µm (1.25 × 16 µm) to 30 µm (1.875 × 16 µm). When Tf = 35°, Fl = 20 µm was achieved (Supplementary Fig. S1a); when Tf = 25°, Fl = 25 µm was achieved (Supplementary Fig. S1b), and when Tf = 10°, Fl = 30 µm was achieved (Supplementary Fig. S1c).

### Device design optimization

Because of the large difference in size between the pairing candidates, it was important to select the correct combination of focusing length and support pillar height, as this directly affects the cell capture rate and pairing efficiency. To clarify the effects of support pillar height and focusing length on cell capture efficiency, it was essential to analyze the amount of fluid guided inside and around the cell capture cups. We carried out simulations via COMSOL Multiphysics® to analyze the change in average fluid flow velocity inside the capture cup and around it. The average fluid flow velocity inside the microconfinement zone was calculated over the 3D cutoff line indicated in green, and the velocity around the microconfinement zone was calculated over the 3D cutoff line shown in red. The average pressure generated at the large-cell capture cup junction was calculated over the 3D cutoff indicated in black (Supplementary Fig. S1g). The three microconfinement designs with decreasing Tf angles are shown in Supplementary Fig. S1(a–c). The velocity gradient profiles of these three designs with decreasing Tf values were derived from COMSOL Multiphysics and are shown in Supplementary Fig. S1d–f. Supplementary Fig. S1h, i shows the changes in fluid flow velocities with respect to the focusing length ‘Fl’ and support pillar-to-total channel height ratio ‘X.’ The inlet fluid flow velocity was kept constant at 10 µl/min.

To achieve higher cell capture efficiencies, there should be increased fluid flow inside and decreased fluid flow outside the microconfinement. Using this principle, we carried out a simulation by varying the cell charging length and ratio X. The fluid flow velocities inside and outside the confinement increased with increasing ratio ‘X’ when the focusing length Fl was 20 µm. However, when the focusing length was increased to 25 µm and 30 µm, the fluid flow inside and outside the confinement zone exhibited different patterns with increasing ‘X’ values. The fluid velocities inside the confinement increased with increasing ‘X’ values from 0.22 to 0.28 but again decreased at 0.33. The flow velocities outside the device decreased with increasing X values from 0.22 to 0.28 and again increased at 0.33. We also analyzed the pressure changes with changes in X values at the capture trap neck over the 3D cutoff line (black). A gradual decrease in pressure was observed with increasing X values from 0.22 to 0.33 (Supplementary Fig. S1j).

Next, we studied the impact of the inlet flow velocity on the fluid flow velocity inside (Supplementary Fig. S1k–m) and outside the microconfinement zone (Supplementary Fig. S1n–p). The flow velocity inside the device increased with increasing flow rate from 1 µl/min to 50 µl/min for ‘X’ values of 0.22 to 0.28 and then decreased to 0.33. The patterns were observed for Fl values of 25 µm and 30 µm. However, for Fl = 20 µm. The flow velocity increased continuously until X = 0.33. The flow velocities outside the confinement did not change at low flow rates of 1 and 10 µl/min but changed at higher flow rates of 25 and 50 µl/min. The fluid flow velocities decreased with increasing ‘X’ values. This trend was observed for Fl = 25 and 30 µm. However, for Fl = 20 µm, the flow velocity increased at higher flow rates (25 and 50 µl/min).

We chose two Fl values of 25 µm and 30 µm and two X values of 0.22 and 0.28 for designing and fabricating the microconfinement; this is mainly due to sustained increased fluid flow inside and decreased fluid flow outside the device.

#### Cell pairing

**Fig. 2:**
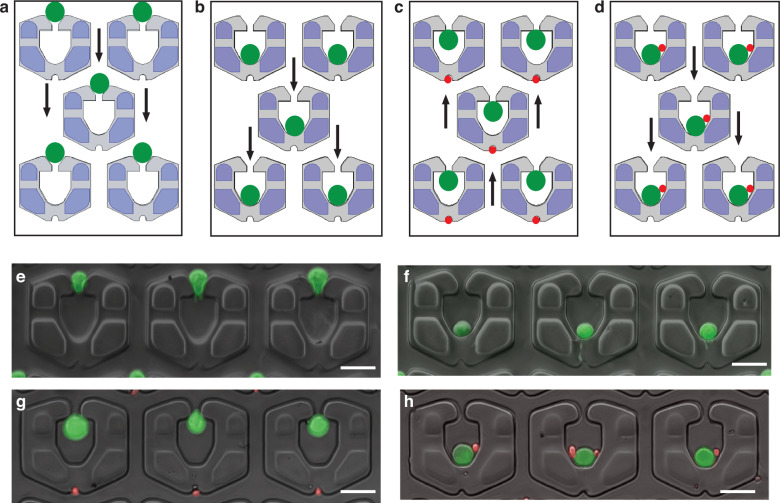
Size selective cell pairing steps of T cell (4 μm) and macrophagepairing (16–20 μm). **a** Step1—Large cell capture on the largecell capture cup; **b** Step 2—Placing large cell into pairing compartment; **c** Step 3—Small cell capture on small cell capture cup; **d** Step 4—Pairingsmall cell and large cell inside the pairing compartment. Images of pairing ofprimary macrophage with OT-1 T cells. **e** Image of capture of primary macrophage cells on the back side capture cup. **f** Placing macrophagecells into the pairing cup via deformation by increasing the backside flow rate. **g** Capturing the OT1 T cells on to the front capture cups. **h** Placingthe T cells into the pairing cup via back flow. The scale bars = 30 μm for (**e**–**h**)

A four-step process was used to capture and pair macrophages with T cells (see Fig. 2). First, the macrophages were loaded on the front-loading chamber and flown into the capture cup Cl at a flow rate of 1 µl/min (Fig. 2a). The flow rate was increased to 50 µl/min to deform and squeeze the cell into the pairing compartment (Fig. 2b) (Supplementary movie M1). Then, the flow direction was reversed (step 3), and T cells were withdrawn from the other side of the device at a flow rate of 1 µl/min and captured onto the Cs (Fig. 2c and Supplementary Movie M2). Finally, the flow direction was reversed.

The flow rate was increased to 10 µl/min to place the T cells in the pairing compartment (Fig. 2d and Supplementary movie M3). The calculation of pairing efficiency (~39%) in the captured video frame is shown in Supplementary Fig. S2a. The images of pairing OT-1 CD8+ T cells with macrophages are shown in Fig. 2e–h.

The microfluidic devices were fabricated according to the dimensions obtained from the simulation data. Microfluidic size-selective pairing experiments were carried out on the fabricated devices. The pairing efficiencies are shown in Supplementary Table T2. The highest pairing efficiency of ~40% was achieved with a 24 µm large cell focusing length, 20 µm small cell focusing length and support pillar-to-total channel height ratio X of 0.28.

**Fig. 3:**
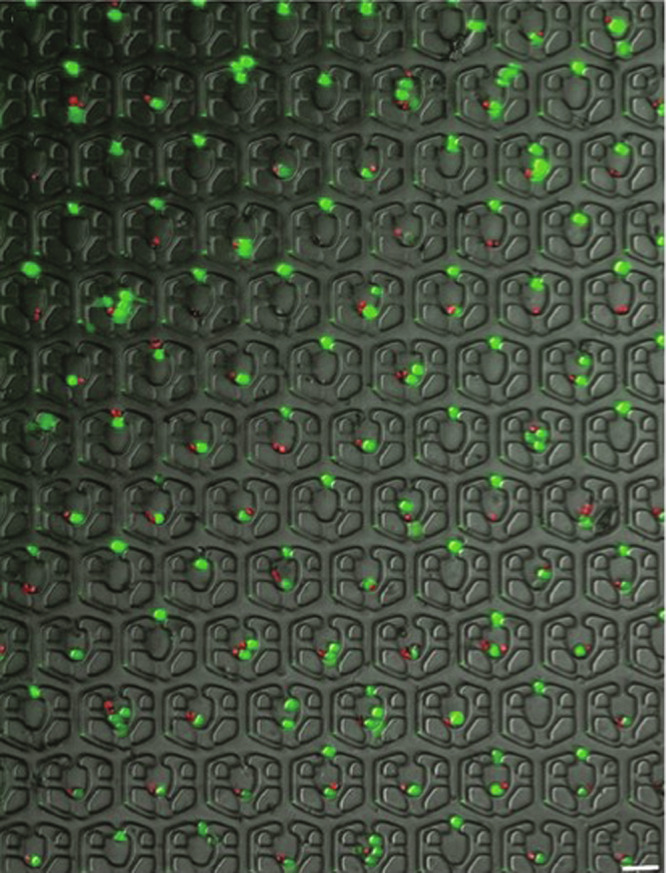
Pairing of macrophages and OT-1 CD8+ T cells at low magnification; scale bar = 40 μm

The images of the paired cells at low magnification are shown in Fig. 3. The calculation of the pairing efficiency (41.66%) is shown in Supplementary Fig. S2b.

### Population-level responses of mouse primary macrophages after LPS and rapamycin treatment

#### Macrophage activation

Three types of treatments were added to the culture media to activate mouse primary macrophages isolated from the bone marrow of 4- to 6-week-old mice. First, we treated macrophages with a combination of rapamycin and LPS (synergistic trigger). We then added rapamycin after the addition of LPS (sequential trigger 1), after which we added rapamycin before the addition of LPS (sequential trigger 2). Here, macrophages were treated with LPS alone, and a culture medium incubated with macrophages was used as a control.

#### Metabolism and survival

We analyzed the effects of these treatments on the metabolism and survival of mouse primary macrophages. As previously reported, LPS induces high glucose metabolism in mouse dendritic cells; this leads to excessive glucose consumption and lactate formation (an end product of glycolysis)^21^. These factors further lead to rapid glucose exhaustion and cell death. However, when rapamycin is introduced into cell culture media, glucose consumption is significantly reduced, leading to survival in mouse DCs^22,23^. To understand whether a similar phenomenon occurs in primary mouse macrophages, we checked the glucose and lactate levels in the cells after 48 h of treatment. We next investigated how the addition of rapamycin and LPS affects metabolism in primary macrophages.

We observed substantial glucose consumption and lactate formation in mouse primary macrophages following LPS treatment. Interestingly, the addition of rapamycin with LPS (synergistic trigger) led to a significant reduction in glucose consumption, as evidenced by higher glucose levels in the culture media, coupled with an increase in lactate production.

Intriguingly, when rapamycin was administered before LPS (sequential trigger 2), we observed a decrease in glucose consumption compared with that of the synergistic treatment, along with a concurrent reduction in lactate production. Conversely, in the case of rapamycin added after LPS (sequential trigger 1), both glucose consumption and lactate production were greater compared to what was observed under the synergistic and sequential trigger 2 scenarios (see Supplementary Fig. S3).

To delve deeper into the impact of these treatments on macrophage survival, we conducted an annexin V FIT-PI assay 96 h posttreatment (see Supplementary Fig. S4). This analysis revealed a significantly greater number of apoptotic and dead cells in the LPS-treated population than in the control population, confirming the findings from the metabolic assays. These findings suggest that macrophages stimulated with sequential trigger 2 exhibit enhanced T-cell activation capacity and prolonged survival, indicating a potential therapeutic advantage in this sequential triggering approach.

#### Costimulatory receptor expression and secretory responses

**Fig. 4:**
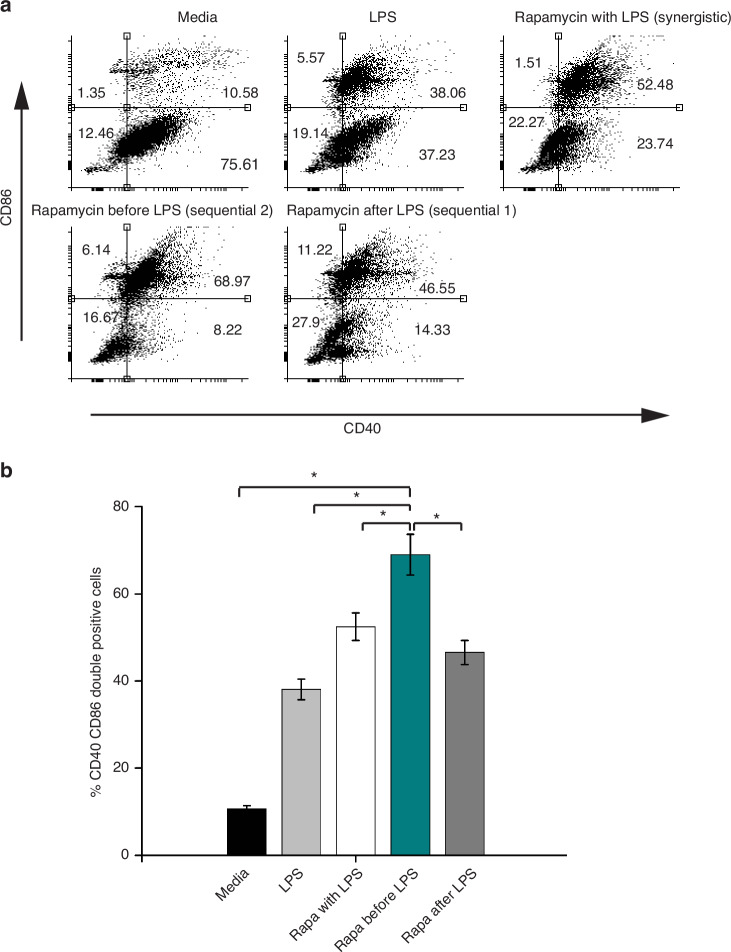
Flow cytometry data recorded after 24 h for costimulatory receptor expression (CD40 and CD86) in primary macrophages activated by synergistic and sequential triggers. **a** FACS dot plots of CD40+ CD86+ cells. **b** Bar plots showing the number of CD40+ CD86+ cells. Significant differences were determined via one-way analysis of variance. *P < 0.05

Costimulatory receptors are among the three signals present on antigen-presenting cells and play pivotal roles in activating T cells upon the establishment of immune synaptic contact. The increased expression of these costimulatory receptors directly correlates with the activation of T cells both in the short and long term. The aim of our experiments was to dissect the expression of costimulatory receptors and the secretion of proinflammatory and anti-inflammatory cytokines from macrophages under three treatment conditions. Flow cytometry analysis (Fig. 4) of the expression of CD86 versus that of CD40 revealed that the highest proportion of macrophages that were positive for both CD86 and CD40 were stimulated sequentially with trigger 2 (68.97%), followed by synergistically triggered cells (52.48%). This proportion decreased sequentially with trigger 1 (46.55%) and LPS-treated cells (38.06%). The untreated cells presented the lowest percentage (10.58%) of cells that were positive for CD86 and CD40.

ELISA data revealed that synergistic and sequential treatments promoted the production of the proinflammatory cytokines TNF-α and IL12p70 but negatively affected the production of the regulatory cytokine IL10 in macrophages. Proinflammatory secretions were negatively regulated, and IL10 secretion was augmented when macrophages were treated with LPS alone. Compared with synergistic and sequential trigger 1 macrophages, sequential trigger 2 activated macrophages presented increased TNF-α and IL12 p70 levels and equivalent IL10 secretion levels. The LPS-treated cells showed significantly increased IL10 secretion (Supplementary Fig. S5).

## Tracking single-cell level responses via the SSP platform

### OT-1 CD8+ T-cell responses after 12 h of macrophage activation

**Fig. 5:**
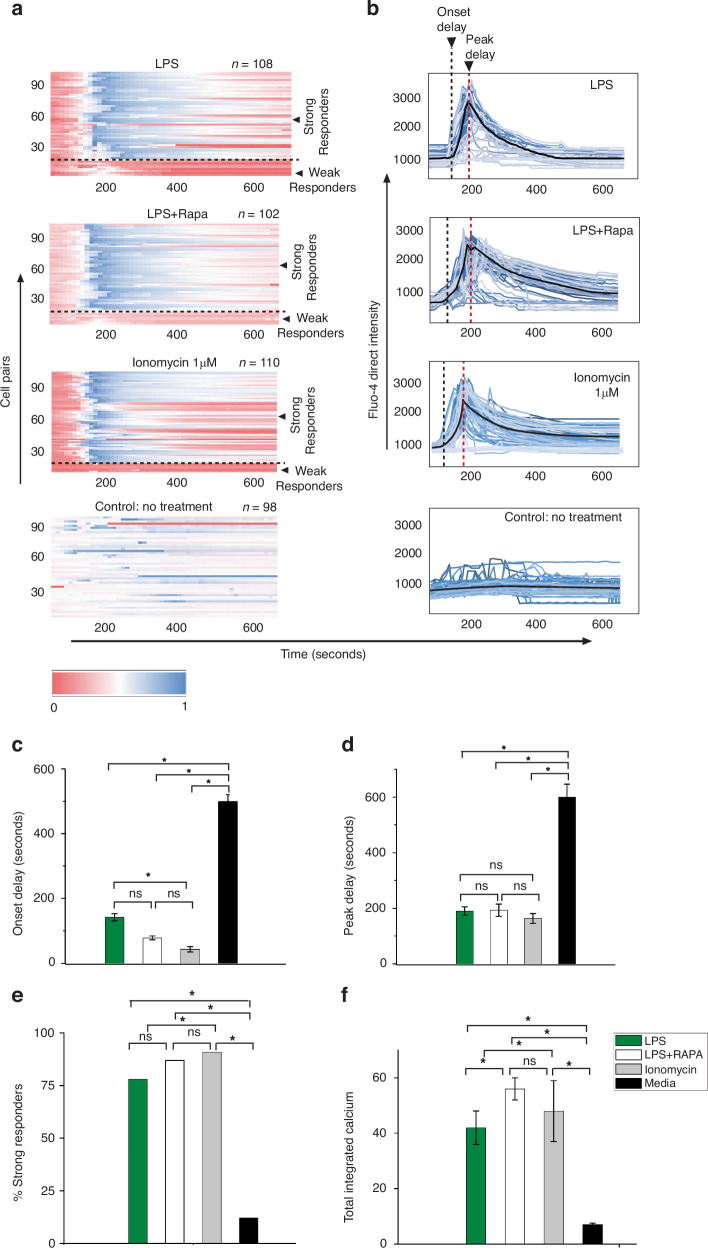
OT-1 T-cell response when paired with macrophages after 6 h of activation. **a** Heatmaps showing time-dependent calcium mobilization in OT-1 T cells after pairing with macrophages treated with LPS, a combination of LPS and rapamycin, PMA+ionomycin for nonspecific activation and the untreated control. **b** Calcium mobilization plots show the onset delay (black dotted line) and peak delay (red dotted line). Comparative analysis of four essential parameters governing the T-cell response after pairing with macrophages 6 h after activation for **c** onset delay, **d** peak delay, **e** total integrated calcium and **f** percentage of strong responders. Significant differences are indicated by “*” and nonsignificant differences are indicated by “ns.” Significant differences were determined with one-way analysis of variance. *P < 0.05

To monitor early T-cell responses in detail, we primed primary macrophages with LPS both with and without rapamycin. We subsequently scrutinized cytosolic calcium fluxes in T cells paired with these activated macrophages. The resulting calcium responses are depicted as heatmaps in Fig. 5a and corresponding intensity plots in Fig. 5b. During the profiling of cytosolic calcium fluxes in T cells after macrophage interaction, we identified four distinct characteristic patterns, as previously documented^24^: onset delay, peak delay, percentage of strong responders, and total integrated calcium levels in T cells.

OT-1 CD8+ T lymphocytes were paired with macrophages 6 h after treatment with the triggering agents. The triggering agents used were LPS, synergistic treatment with LPS and rapamycin, PMA with ionomycin (a nonspecific triggering agent) and culture media as a control.

While there was no significant difference in onset delay (Fig. 5c) observed in T cells paired with LPS-treated and synergistically activated macrophages, those activated by PMA with ionomycin presented a significantly shorter onset delay than those in the LPS-treated group.

The peak delay levels (Fig. 5d) in OT-1 CD8+ T cells paired with LPS, synergistically activated macrophages, and PMA with ionomycin-treated macrophages were slightly different; however, all three groups significantly differed from the medium-treated macrophage control group.

**Fig. 6:**
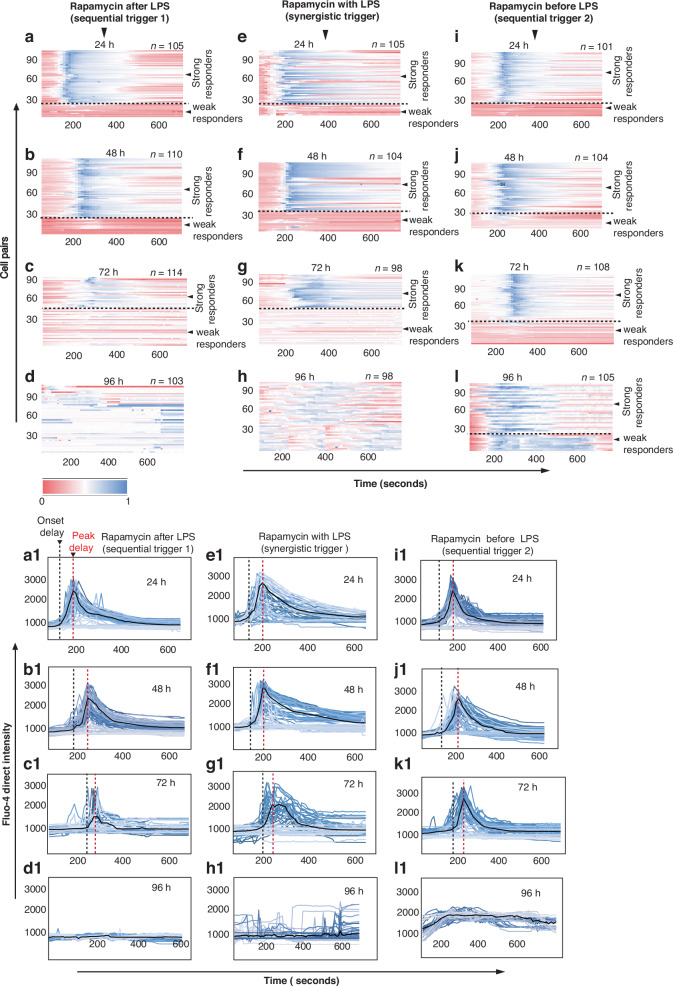
Heatmaps and intensity plots of T-cell calcium signaling responses against sequentially and synergistically activated macrophages from 24 to 96 h of activation. **a**–**d** Heatmaps of the T-cell calcium response after contact with macrophages activated by sequential trigger 1, **e**–**h** synergistic trigger, or **i**–**l** sequential trigger 2. **a1**–**d1** Intensity plots of the responses corresponding to heatmaps (**a–d**). **e1**–**h1** Intensity plots of the responses corresponding to heatmaps (**e**–**h**). **i1**–**l1** Intensity plots of the responses corresponding to heatmaps (**i**–**l**)

Furthermore, the total integrated calcium levels (Fig. 6e) were notably greater in the PMA with ionomycin-treated group than in the LPS-treated, synergistically activated, and control groups. We used a K-means clustering algorithm to segregate weak and strong responders on the basis of their total integrated calcium values.

The percentage of strong responders (Fig. 5f) was most pronounced in the PMA with ionomycin-treated group, with no significant difference observed between the LPS-treated and synergistically activated groups. Notably, all three treatment groups significantly differed from the media-treated control group.

### Comparison of the responses of OT-1 CD8+ T cells paired with sequentially and synergistically triggered macrophages in combination with rapamycin in the presence of TLR agonists

To comprehensively assess the enduring effects of treatment on macrophages and their consequential influence on T-cell fate, we conducted a 96-h study (see Fig. 6). Macrophages underwent maturation within cell culture flasks via three distinct triggering methods.

The first method involved sequential trigger 1, wherein macrophages were exposed to LPS for 4 h, followed by the addition of rapamycin. Sequential trigger 2 entailed the reverse sequence, with macrophages first treated with rapamycin for 4 h before exposure to LPS. The synergistic trigger involved the simultaneous addition of rapamycin and LPS when culture was initiated. Notably, in all triggering strategies, ovalbumin peptide (OVA (257–264), SIINFEKL) was introduced at the onset of macrophage culture.

The macrophages were then collected from the cell culture flasks after 24, 48, 72 and 96 h of maturation and paired with OT-1 CD8+ T cells inside the microfluidic chip through a size-selective pairing method as described earlier. We compared the onset delay, peak delay, percentage of strong responders, and total integrated calcium and IFN-γ secretions in these T cells.

**Fig. 7:**
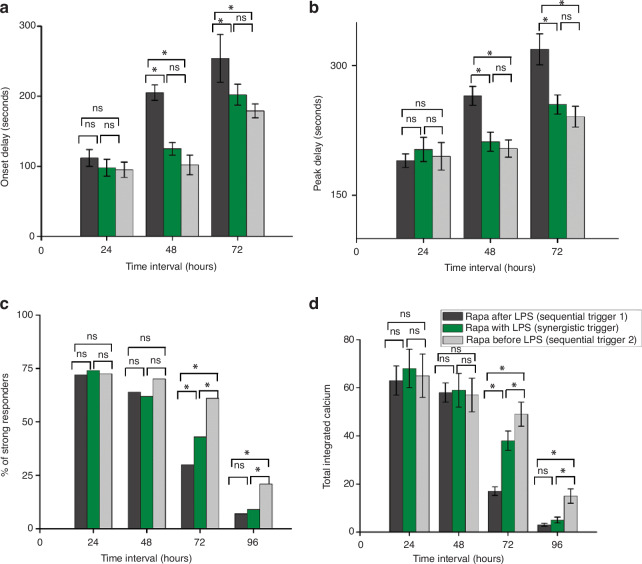
Comparison of calcium signaling responses in T cells after pairing with macrophages triggered sequentially and synergistically with rapamycin and LPS. **a** Onset delay, **b** peak delay, **c** percentage of strong responders, and **d** total integrated calcium; ns = not significant, * = significant. Significant differences were determined via one-way analysis of variance. *P < 0.05

As depicted in Fig. 7, the temporal dynamics of onset and peak delay responses in OT-1 CD8+ T cells exhibited no discernible disparity within the initial 24 h of macrophage activation. However, upon pairing 48 and 72 h after macrophage activation, a notable trend emerges: T cells coupled with macrophages activated by sequential trigger 1 exhibit markedly greater onset and peak delay responses than those activated by sequential trigger 2 and the synergistically triggered group.

Throughout the first 24 and 48 h, there is no appreciable distinction in the percentage of strong responders. However, at the 72- and 96-h time points, compared with those in the other two groups, the number of strongly responsive T cells in the second group was substantially greater. Interestingly, at these later time points, the quantity of strong responders among T cells triggered by sequential trigger 1 mirrors that of synergistically activated cells.

Moreover, the assessment of integrated calcium in T cells revealed no discrepancy after 24 and 48 h. However, T cells triggered by sequential trigger 2 presented a significant elevation in integrated calcium levels during the 72- and 96-h intervals than did those activated by the other two methods. Intriguingly, macrophages activated by sequential trigger 1 triggered a more rapid decrease in cytosolic calcium levels within T cells after 72 h than synergistically triggered cells did.

### Comparison of IFN-γ secretion by T cells activated by sequential and synergistic triggers

**Fig. 8:**
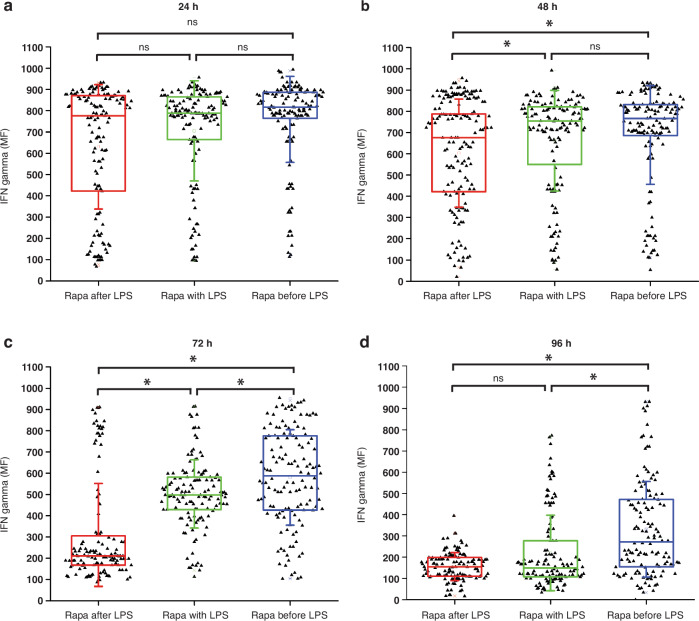
Comparisons of IFN-γ secretion by OT-1 C8+ T cells paired with macrophages inside the chip after. **a** 24 h, **b** 48 h, **c** 72 h, and **d** 96 h, ns = not significant; * = significant. Significant differences were determined via one-way analysis of variance. *P < 0.05

The IFN secretion of T cells was meticulously analyzed within the microfluidic device (Fig. 8), with each dataset comprising 150 to 200 cells. Initially, no discernible difference in IFN secretion was noted among the paired T cells after 24 h of macrophage activation. However, after 48 h, a significant decrease in IFN levels was observed in T cells paired with macrophages activated by sequential trigger 1.

By the 72-h time point, macrophages activated by sequential trigger 2 elicited notably higher IFN levels, contrasting sharply with the lowest IFN levels induced by those activated by sequential trigger 1. Strikingly, at the 96-h time point, T cells paired with macrophages activated by sequential trigger 1 and the synergistic trigger displayed a complete decrease in IFN levels. In stark contrast, those paired with macrophages activated by sequential trigger 2 exhibited a substantial and significant increase in IFN secretion.

### Changes in IFN-γ levels in T cells with changes in cytosolic Ca^2+^ levels

**Fig. 9:**
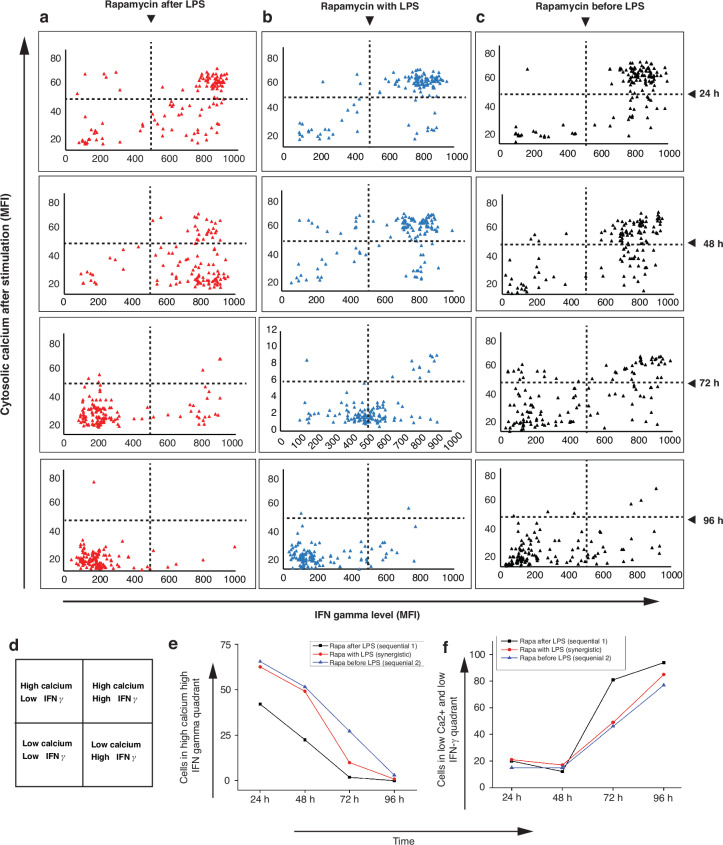
Variation in IFN-γ with the cytosolic calcium level. **a** Response of T cells paired with macrophages activated by the addition of rapamycin after LPS (sequential trigger 1); **b** response to rapamycin with LPS (synergistic trigger); **c** response to rapamycin after LPS (sequential trigger 2); **d** quadrant-wise segmentation of the T-cell response with respect to the quantity of cytosolic calcium and IFN-γ secretions shown in panels (**a**–**c**); **e** plot of the quantification of the change in the number of T cells with high Ca^2+^ and high IFN-γ levels (top right quadrant) at 24-h intervals; **f** plot of the quantification of the change in the number of T cells with low Ca^2+^ and low IFN-γ levels (bottom left quadrant) at 24-h intervals. “*” indicates a significant difference. Significant differences were determined with one-way analysis of variance. *P < 0.05

We examined the interplay between IFN-γ secretion and cytosolic Ca^2+^ levels in T cells (Fig. 9a–c) under the influence of sequentially and synergistically activated macrophages within our microconfinement environment. Figure 9d shows the division of each IFN-γ vs. cytosolic calcium plot into four quadrants, delineating responses on the basis of high or low levels of Ca^2+^ and IFN-γ: high Ca^2+^ and high IFN-γ (top right), high Ca^2+^ and low IFN-γ (top left), low Ca^2+^ and high IFN-γ (bottom right), and low Ca^2+^ and low IFN-γ (bottom left).

We observed that the population of T cells in the high-IFN-γ and high-Ca^2+^ quadrant declined more rapidly over time when paired with macrophages activated by sequential trigger 1, whereas the most enduring population was evident among those activated by sequential trigger 2 (Fig. 9e). Additionally, after 48 h, the number of T cells in the low-Ca^2+^ and low-IFN-γ quadrant increased more rapidly in T cells paired with macrophages triggered by sequential trigger 1 than in those activated by synergistic and sequential trigger 2 (Fig. 9f). These findings suggest that macrophages activated by sequential trigger 2 can sustain T-cell activation for a longer duration, leading to heightened cytotoxic capacity.

## Discussion

Here, we introduce a novel size-selective single-cell pairing platform capable of pairing cells that differ in size by up to fivefold. Our device offers a distinct advantage, facilitating the pairing of single cells while enabling precise triggering with soluble reagents at high spatiotemporal resolution. Crucially, our device allows seamless monitoring of cell performance without sacrificing cell tracking. This innovative approach affords the generation of diverse microenvironmental conditions akin to those encountered within lymph nodes during cellular interactions, an accomplishment unattainable through conventional culture methods.

With 1000 traps per device, we achieved a pairing efficiency of up to 40%. Compared with earlier methods, our device has a lower pairing efficiency, but because our device has a higher trap density per unit area, our device offers over 30% greater pairing efficiency in the same area. Although we investigated the pairing of T cells and macrophages, which differ in size by up to fivefold, the pairing of other cell types with larger differences in size remains to be explored.

Leveraging our platform, we investigated the response of primary mouse macrophages to strategically controlled soluble inputs, assessing their impact on T-cell activation. We investigated, at the single-cell level, the potential of combining rapamycin and Toll-like receptor (TLR) agonists in promoting sustained macrophage activation to increase T-cell functionality. To this end, we explored three distinct combinations of rapamycin and the TLR4 agonist LPS: synergistic (rapamycin with LPS), sequential trigger 1 (rapamycin post-LPS), and sequential trigger 2 (rapamycin pre-LPS).

To our surprise, analysis of single-cell data obtained from the chip revealed that macrophages triggered by sequential trigger 2 presented increased cytosolic calcium levels and faster response times than those triggered by sequential trigger 1 and the synergistic trigger, as evidenced by shorter initiation and peak delay timings in individual T cells. Notably, the superiority of sequential trigger 2 was most pronounced when T cells were paired following 48 h of macrophage activation. Sequential trigger 1, in contrast, resulted in a swifter decline in response time and integrated calcium levels than did the synergistic trigger, a trend mirrored in IFN-γ secretion levels within activated T cells.

**Fig. 10:**
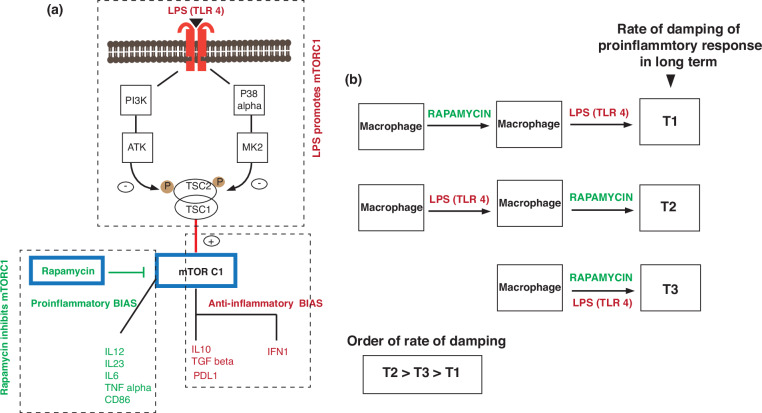
Influence of synergistic and sequential stimulation on macrophage activation and the rate of immune response dampening. **a** LPS influences the activation of the mTORC1 complex, leading to an anti-inflammatory response. Rapamycin inhibits the mTORC1 complex, leading to a proinflammatory response. **b** The sequence of the addition of rapamycin and LPS to macrophages modulates the rate at which the proinflammatory response is dampened in macrophages in the long term. The slowest dampening rate (T1) is shown by macrophages stimulated with rapamycin followed by LPS. The fastest dampening occurs when LPS is added before rapamycin (T2)

Consistently elevated levels of costimulatory receptor expression in macrophages triggered by sequential trigger 2 correlated with heightened T-cell activation even after 72 h. This effect may be attributed to rapamycin-mediated inhibition of mTORC1-mediated signaling pathways, fostering a proinflammatory macrophage phenotype characterized by increased secretion of cytokines, such as IL-12 and TNF-α, and increased costimulatory receptor expression (CD86 and CD40). Conversely, LPS-mediated activation of the mTORC1 pathway, facilitated by the PI3K and p38α signaling cascades, culminated in the release of inhibitory cytokines, such as IL-10 (Fig. 10a)^17–19^, as validated by our experimental findings in Supplementary Fig. S5c.

These results led us to hypothesize that early rapamycin administration counteracts the inhibitory effects of LPS-induced mTORC1 activation, with the anti-inflammatory impact being particularly pronounced when LPS precedes rapamycin administration, leading to rapid attenuation of T-cell responses after 48 h of macrophage activation (Fig. 10b). Furthermore, the sustained predominance of the effect of rapamycin, even following LPS administration, underscored the subtle but significant alterations in response dynamics, which were notably evident after the initial 24 h, with pronounced shifts in calcium response profiles and IFN-γ levels.

Although the more pronounced effect of sequential trigger 2 is evident via our single-cell experiments, the outcome of this treatment method should be explored through meticulous in vivo experiments to establish this in therapy.

## Conclusions

In conclusion, our developed microfluidic device represents an advancement in the realm of single-cell analysis, particularly in elucidating interactions across diverse cell types with substantial variations in size. Unlike traditional methods reliant solely on photographic documentation, our device enables real-time assessment, which is crucial for understanding dynamic cellular events. While our device has a lower pairing efficiency than that of previous methods for pairing cells with size differences, its higher trap density translates to increased throughput per unit area, increasing its utility for research and therapeutic applications. However, the full potential of our device remains untapped beyond that of macrophages and T cells, warranting exploration across a broader spectrum of cell types, especially those differing significantly in size.

Through our microfluidic platform, we uncovered the intricate dynamics of T-cell responses to macrophages, revealing the profound impact of the rapamycin administration sequence on therapeutic outcomes. Surprisingly, administration of rapamycin before LPS to macrophages resulted in prolonged T-cell activation, a slower decrease in the proinflammatory response, increased expression of costimulatory receptors, and an extended lifespan in primary macrophages. While these single-cell findings suggest improved T-cell responses, further preclinical experiments are imperative to validate therapeutic efficacy.

Ultimately, our work holds great promise for pioneering cutting-edge immunotherapeutic interventions targeting cancer and various immune-related disorders, propelling advancements in personalized medicine and enhancing patient outcomes.

## Materials and methods

### Materials

PDMS (Sylgard 184) was procured from Dow Corning, USA. The Fluo-4 direct calcium tracking dye was procured from Thermo Fisher Scientific (USA). Anti-mouse IFN-γ antibodies were procured from Cell Signaling Technology (USA). The SU8 2002 and SU8 2010 photoresists were procured from Microchem Corporation (USA). Anti-CD 86 PE and anti-CD40 FITC antibodies were procured from eBioscience (Thermo Fisher Scientific).

### Device fabrication

Microfluidic devices were fabricated by first creating the molds using two different SU8 photoresists procured from Microchem Corporation on optically flat silicon substrates via a three-step lithography process. First, a 2-μm-thick adhesion layer using SU8 2002 was created via blank UV exposure via a transparent mask glass plate. The 3–5-μm-thick support pillars were then created via SU8 2002 and SU8 2005, followed by UV exposure. The third layer of the capture cups was created by spin coating a 10–12-μm-thick layer of SU8 2010, followed by UV exposure. After mold fabrication, a 3-mm-thick PDMS layer (Sylgard 184, Dow Corning) was poured over the SU8 molds and cured overnight at 80 °C. The PDMS chips were then peeled off and punched to create inlet holes for tubing connections. The chips were subsequently bonded to cover slips via the generation of oxygen plasma (Harrick plasma system). The bonded chips were further cured at 80 °C for 2 h after the steel pins were inserted and pins were sealed with PDMS to prevent leakage.

### Primary mouse macrophage culture

Primary macrophages from the bone marrow of 6-week-old male OT1 mice were harvested. The isolated bone marrow cells were cultured in DMEM supplemented with 10% FBS and 1% penicillin-streptomycin (25 ng/ml). M-CSF was added to the media, and the cells were differentiated into primary macrophages for 7 days. The nonadherent cells were removed from the 6-well culture plate to obtain differentiated primary macrophages that remained adherent in the culture plate^25^.

### Primary OT-1 CD8+ T-cell isolation and culture

OT-1 T cells were isolated from the spleens of the same mice. A magnetic activated cell sorter from Meltenyi Biotech was used to positively select OT-1 CD8+ T cells^26^. The isolated cells were cultured in RPMI 1640 media supplemented with HEPES buffer, L glutamine and 10% fetal calf serum (FCS).

### Treatment protocol for maturation of macrophages

The differentiated macrophages were transferred to 24-well plates and matured by the addition of LPS or rapamycin in combination. LPS (15 ng/ml) was added to freshly adhered macrophages, which were then cultured for 12 and 48 h in 6-well plates. A combination of 15 ng/ml LPS and 25 ng/ml rapamycin was added to the macrophages, which were subsequently cultured for the same amount of time. These cells were then added to microfluidic chips for pairing with T cells.

### Glucose and lactate assays

Glucose and lactate assays were performed on primary macrophages independently of the microfluidic device. The macrophages were cultured in high-glucose DMEM for 48 h in the presence or absence of different maturation reagents as described above. The glucose and lactate concentrations in the media were analyzed via glucose and lactate assay kits from Sigma Aldrich. Both the glucose and lactate assays require 50 µl of sample for each reaction (well) and 50 µl of reaction master mix (supplied by the manufacturer). After the sample was mixed with the reaction mixture, the plate was incubated for 30 min at 37 °C, after which the absorbance was measured at 570 nm. The unknown sample concentration value was obtained from the standard curve.

### Costimulatory receptor expression

After activation, primary macrophages were treated with monoclonal antibodies against CD40 and CD86 for 30 min. The unbound antibodies were removed by washing with ice-cold PBS. The expression of these genes was analyzed via flow cytometry.

### Inflammatory cytokine quantification via flow cytometry

Primary macrophage supernatants were analyzed for TNF-α, IL-12p70 and IL-10 by flow cytometry via a bead array mouse inflammation kit (BD Bioscience).

### Microfluidic device setup

The device was made bubble-free with 70% ethanol, then the device surface was blocked with 7% BSA for 1 h before loading the cells. The device had a single inlet and a single outlet. One end of the device was connected to a positive pressure source for cell flow into the device and a negative pump for withdrawing fluid connected to the reservoir and a valve splitter for switching in the low direction.

The larger cells were loaded in the 15 ml Falcon cell reservoir, and the smaller cells were loaded in the chip reservoir. The larger cells diluted to 1 × 10^5^ cells/ml were pipetted directly into the loading chamber and pushed into the device, followed by a brief washing step to remove uncaptured cells. The small cells then flowed through the other inlet by switching the flow direction and were captured on the small cell cup. This was then followed by capturing the small cell via flow reversal into the pairing compartment. The device was placed on a laser scanning confocal microscope (FV 3000) for imaging experiments. A schematic of the setup is provided in Supplementary Fig. S6.

### On-chip calcium flux study

The staining of T cells was carried out via the nonratiometric calcium indicator dye Fluo 4, which was obtained directly from Invitrogen. A Fluo-4 direct stock solution was prepared by adding 10 ml of Fluo-4 Direct™ calcium assay buffer and 200 µl of 250 mM probenecid stock solution to 1 ml of Fluo-4 Direct™ calcium reagent. The T cells were cultured in a 96-well plate. In each well, 50 µl of the Fluo-4 direct stock mixture was added to 50 µl of phenol red-free cell culture media for 1 h at 37 °C and 5% CO_2_ inside a cell culture incubator. The cells were then injected into the chip for pairing with the activated macrophages. FLUOVIEW software was used to acquire the data. Regions of interest (ROIs) were selected at ×10 magnification. The data were recorded after the background was removed.

### On-chip quantification of intracellular IFN-γ levels in T cells

The primary naïve OT-1 CD8+ T cells were initially cultured in phenol red-free RPMI media and primed with IL2. The cells were then treated with a 1 µl/ml Golgi plug from Invitrogen before being placed and paired with primary activated macrophages. After completion of the pairing experiments, the cells were cultured inside the chip for 5 h. This was followed by on-chip fixation with 4% paraformaldehyde for 15 to 20 min, followed by permeabilization of the cells with 0.5% Triton X-100. The cells were then blocked with PBS containing 10% bovine serum, 0.5% Triton X-100, and 1% BSA for 30 min. The cells were then stained with anti-mouse IFN-γ antibodies (BD Pharmingen) inside the chip suspended in a blocking buffer. The chip was incubated overnight at 4 °C and then washed in PBS before analysis via fluorescence microscopy.

### Measurement strategies for Ca^2+^ and IFN-γ in T cells

Calcium oscillations and IFN-γ were measured in T cells in a stepwise manner. The membrane permeabilization steps for IFN-γ measurement were performed after the calcium response in T cells was measured. First, the cells were treated with Fluo-4 directly, after which they were loaded onto the chip for pairing with macrophages and tracking the calcium response. After the calcium response was measured, the T cells were permeabilized and strained with an IFN-γ antibody to measure the levels of IFN-γ.

### Annexin V-FITC-PI assay

An Annexin V-FITC early apoptosis assay was performed on treated macrophages via a detection kit from Cell Signaling Technology. After the macrophages were cultured in a cell well culture plate with different treatments for 96 h in DMEM, the cells were washed in ice-cold PBS, and 2 × 105 cells were aliquoted from the assay mixture, after which 96 µl of 1× Annexin V binding buffer was added. One microliter of Annexin V-FITC conjugate and 12.5 μl of propidium iodide (PI) solution were added to a 96 µl aliquot. This mixture was incubated for 10 min at room temperature in the dark. The solution was then diluted in 250 µl of ice-cold, 1X Annexin V binding buffer, and flow cytometry experiments were performed. The results of the experiment are reported in Supplementary Fig. S4.

### Collection of calcium response data

T cells were first mixed with Fluo-4 direct dye from Invitrogen in complete phenol red-free media at a 1:1 ratio at 37 °C for 1 h. The staining of T cells was carried out via the nonratiometric Fluo 4 direct calcium indicator dye(Invitrogen). The complete phenol red-free media was mixed with the dye at a 1:1 ratio and incubated for 1 h at 37% and 5% CO_2_ inside a cell culture incubator. The cells were then injected into the chip for pairing with the activated macrophages. FLUOVIEW software was used to acquire the data region of interest (ROI), which was selected at ×10 magnification, where each ROI represented a single cell. In the ×10 magnification range, we could select up to 40 different ROIs for collecting the data. For each experiment, we selected 8 to 10 different ROIs to obtain 1 set of calcium responses. All the data were collected after background removal.

### Calcium response data analysis

The calcium oscillations were analyzed by clustering the data via the k-means clustering algorithm. Here, the sq Euclidean distance was used as a distance measure. The quality and number of clusters were obtained by optimizing the average silhouette scores (Supplementary Fig. 7). The data were scaled and plotted via scripts in R to segregate the weak and strong responders. The recorded data were then analyzed via custom-written scripts in R, and the final data were plotted after background removal. The differences in IFN-γ responses among the different groups were analyzed via one-way ANOVA, and the means were compared via Tukey’s test.

## Supplementary information

Supplementary material

Supplementary movie M1

Supplementary movie M2

Supplementary movie M3

## References

[1] Reijnders, J. H. P. Strong signals from streptavidin–biotin. *Nature* **324**, 2 (1986). 10.1038/320557a0.

[2] Wang, S. & Lee, L. J. Micro-/nanofluidics based cell electroporation. *Biomicrofluidics* **7**, 011301 (2013). 10.1063/1.4774071 PMC3555966 23405056

[3] Bakker Schut, T. C. et al. Selective electrofusion of conjugated cells in flow. *Biophys. J.* **65**, 568–572 (1993). 8218887 10.1016/S0006-3495(93)81128-7 PMC1225759

[4] Wu, C. et al. A planar dielectrophoresis-based chip for high-throughput cell pairing. *Lab Chip* **17**, 4008–4014 (2017). 29115319 10.1039/c7lc01082f

[5] Felton, E. J., Copeland, C. R., Chen, C. S. & Reich, D. H. Heterotypic cell pair co-culturing on patterned microarrays. *Lab Chip* **12**, 3117–3126 (2012). 22739471 10.1039/c2lc40349h PMC3444241

[6] Collins, D. J. et al. Two-dimensional single-cell patterning with one cell per well driven by surface acoustic waves. *Nat. Commun.* **6**, 1–11 (2015). 10.1038/ncomms9686 PMC4659840 26522429

[7] Guo, F. et al. Three-dimensional manipulation of single cells using surface acoustic waves. *Proc. Natl Acad. Sci. USA* **113**, 1522–1527 (2016). 26811444 10.1073/pnas.1524813113 PMC4760790

[8] Arai, F. et al. On chip single-cell separation and immobilization using optical tweezers and thermosensitive hydrogel. *Lab Chip* **5**, 1399–1403 (2005). 16286972 10.1039/b502546j

[9] Li, L. et al. A controllable, centrifugal-based hydrodynamic microfluidic chip for cell-pairing and studying long-term communications between single cells. *Anal. Chem.* **91**, 15908–15914 (2019). 31741379 10.1021/acs.analchem.9b04370

[10] Li, W. H. L., Wang, H., Huang, L. & Michael, S. A. High-throughput deterministic pairing and coculturing of single cells in a microwell array using combined hydrodynamic and recirculation flow captures. *Biomicrofluidics* **26**, 15 (2021). 10.1063/5.0066668 PMC8550803 34737839

[11] Skelley, A. M., Kirak, O., Suh, H., Jaenisch, R. & Voldman, J. Microfluidic control of cell pairing and fusion. *Nat. Methods* **6**, 147–152 (2009). 19122668 10.1038/nmeth.1290 PMC3251011

[12] Dura, B., Liu, Y. & Voldman, J. Deformability-based microfluidic cell pairing and fusion. *Lab Chip* **14**, 2783–2790 (2014). 24898933 10.1039/c4lc00303a

[13] Shaik, F. A. et al. Pairing cells of different sizes in a microfluidic device for immunological synapse monitoring. *Lab Chip* **22**, 908–920 (2022). 35098952 10.1039/d1lc01156a

[14] Cheever, M. A. & Higano, C. S. PROVENGE (Sipuleucel-T) in prostate cancer: the first FDA-approved therapeutic cancer vaccine. *Clin. Cancer Res.* **17**, 3520–3526 (2011). 21471425 10.1158/1078-0432.CCR-10-3126

[15] Kim, J. H. et al. Enhancement of DC vaccine potency by activating the PI3K/AKT pathway with a small interfering RNA targeting PTEN. *Immunol. Lett.* **134**, 47–54 (2010). 20727912 10.1016/j.imlet.2010.08.008

[16] Kim, J. H. et al. Enhancement of dendritic cell-based vaccine potency by anti-apoptotic siRNAs targeting key pro-apoptotic proteins in cytotoxic CD8+ T cell-mediated cell death. *Immunol. Lett.* **122**, 58–67 (2009). 19135479 10.1016/j.imlet.2008.12.006

[17] Weichhart, T. et al. The TSC-mTOR signaling pathway regulates the innate inflammatory response. *Immunity* **29**, 565–577 (2008). 18848473 10.1016/j.immuni.2008.08.012

[18] Haidinger, M. et al. A versatile role of mammalian target of rapamycin in human dendritic cell function and differentiation. *J. Immunol.* **185**, 3919–3931 (2010). 20805416 10.4049/jimmunol.1000296

[19] Fukao, T. et al. P13K-mediated negative feedback regulation of IL-12 production in DCs. *Nat. Immunol.* **3**, 875–881 (2002). 12154357 10.1038/ni825

[20] Amiel, E. et al. Inhibition of mechanistic target of rapamycin promotes dendritic cell activation and enhances therapeutic autologous vaccination in mice. *J. Immunol*. **189**, 2151–2158 (2012). 10.4049/jimmunol.1103741 PMC3424310 22826320

[21] Krawczyk, C. M. et al. Toll-like receptor–induced changes in glycolytic metabolism regulate dendritic cell activation. *Blood* **115**, 6–8 (2010). 10.1182/blood-2009-10-249540 PMC2890190 20351312

[22] Csibi, A. & Blenis, J. Appetite for destruction: the inhibition of glycolysis as a therapy for tuberous sclerosis complex-related tumors. *BMC Biol.* **9**, 69 (2011). 22018140 10.1186/1741-7007-9-69 PMC3198763

[23] Düvel, K. et al. Activation of a metabolic gene regulatory network downstream of mTOR complex 1. *Mol. Cell.* **39**, 171–183 (2010). 20670887 10.1016/j.molcel.2010.06.022 PMC2946786

[24] Dura, B. et al. Profiling lymphocyte interactions at the single-cell level by microfluidic cell pairing. *Nat. Commun.* **6**, 5940 (2015). 25585172 10.1038/ncomms6940

[25] Mendoza, R. et al. Mouse bone marrow cell isolation and macrophage differentiation. *Methods Mol. Biol.* **2455**, 85–91 (2022). 35212988 10.1007/978-1-0716-2128-8_8 PMC8936184

[26] Bayne, L. J. et al. Tumor-derived granulocyte-macrophage colony-stimulating factor regulates myeloid inflammation and T cell immunity in pancreatic cancer. *Cancer Cell* **21**, 822–835 (2012). 22698406 10.1016/j.ccr.2012.04.025 PMC3575028

