# Supplementary material for: Size-selective microfluidics delineate the effects of combinatorial immunotherapy on T-cell response dynamics at the single-cell level
Source: Microsyst Nanoeng. 2024 Nov 26;10:178. doi: 10.1038/s41378-024-00769-3 (PMC11589710; doi:10.1038/s41378-024-00769-3)
Supplement: Supplementary file 1 — Supplementary material [file 41378_2024_769_MOESM1_ESM.docx]

**Supplementary Information**

**Size-selective microfluidics delineate effects of combinatorial immunotherapy on T-cell response dynamics at single-cell level**

Ayan Chatterjee, Aniket Bandyopadhyay, Tapas Kumar Maiti, Tarun Kanti Bhattacharyya

**Supplementary files include:**

**Supplementary Figures S1 to S7**

**Supplementary Tables T1 to T2**

**Supplementary Movies M1 to M3**

| Figure S1 | Optimization of device dimensions |
| --- | --- |
| Figure S2 (a) | Calculation of pairing efficiency with respect to supplementary video 3. |
| Figure S2 (b) | Calculation of pairing efficiency with respect to figure 4(e) |
| Figure S3 | The effect of rapamycin treatment in primary mouse macrophages on glucose metabolism and lactate formation in presence of LPS |
| Figure S4 | Annexin V FITC- PI Assay after 96 hours of treatment for analysis number of Apoptotic and dead macrophages. |
| Figure S5 | Costimulatory receptor expressions and cytokine pro-inflammatory and regulatory secretions of primary macrophages after treatment with LPS, LPS with rapamycin and after rapamycin |
| Figure S6 | Schematic of setup of microfluidic chip operations |
| Figure S7 | Determination of average silhouette scores in K means clustering of calcium responses in T cells. |

| Table T1 | Ratios of support pillar height to total channel height |
| --- | --- |
| Table T2 | Pairing efficiencies with respect to varying FL and X values. |

| Movie M1 | Macrophage Capture  . |
| --- | --- |
| Movie M2 | T cell Capture |
| Movie M3 | T cell macrophage pairing |

**
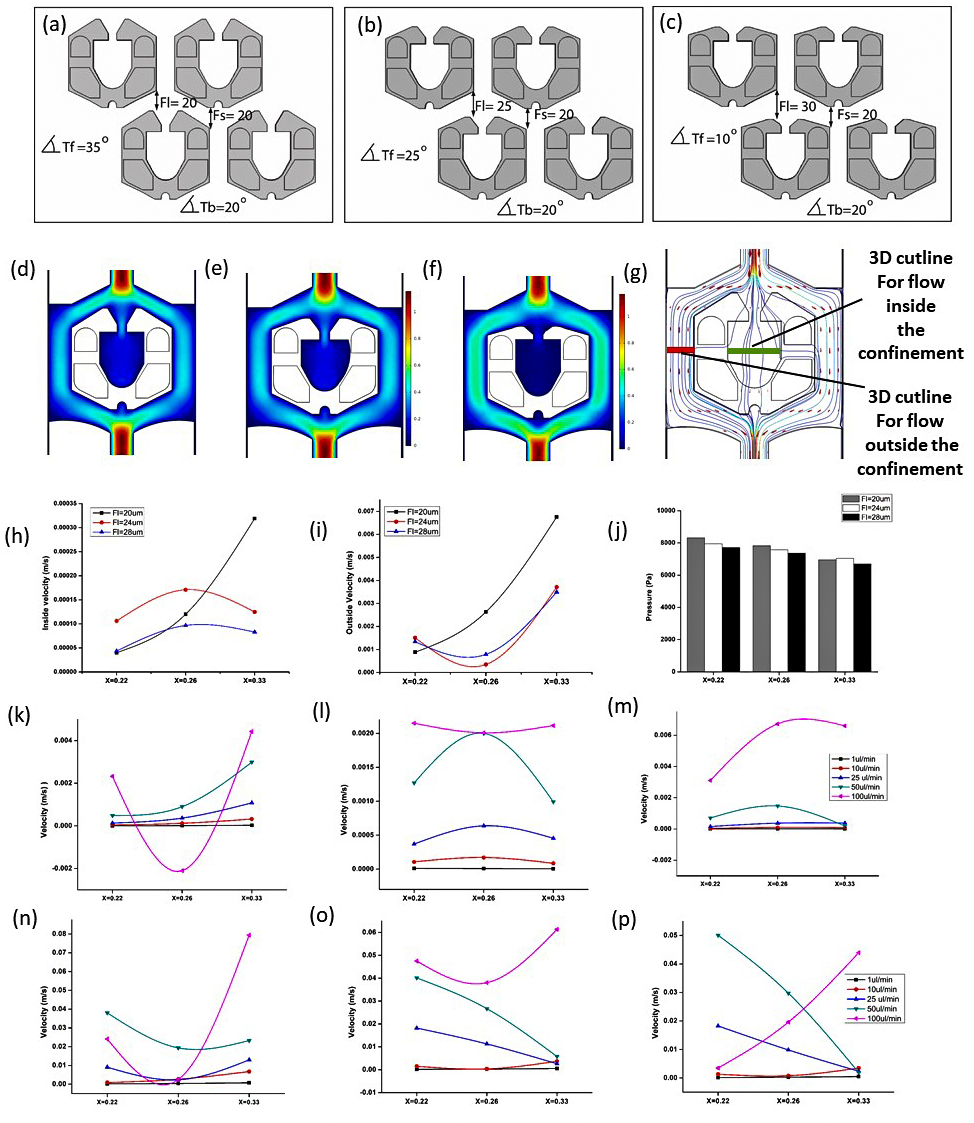
**

**Supplementary figure S1. Optimization of device dimensions** (a- c) Variation of focusing length Fl (25 um ,25 um and 30 um) and tapering angle Tf (35 °, 25° and 10 °) (d)Velocity and Pressure gradient simulations for Fl=20, Tf=35°, (e) Fl=25, Tf=25° and (f) Fl=30, Tf=10 ° (g) The average velocity inside the confinement measured across the 3D cut line line shown in green and average velocity around the device measured across the 3d cut line shown in blue. The Average pressure at the large cell capture junction in measured across 3d cut line shown in black (h) Velocity inside the micro-confinement with 10ul/min inlet velocity with change in Fl (i) Velocity outside the micro-confinement with 10ul/min inlet velocity with change in Fl (j) Variation of pressure at large cell capture inlet junction ( across 3d cut line shown in black ) with change in Fl at 10 ul /min inlet velocity .( k-m) Variation of velocity inside the confinement with change in X value at different flow rates with constant Fl values (n-p) Variation of velocity outside the confinement with change in X value at different flow rates with constant Fl values.


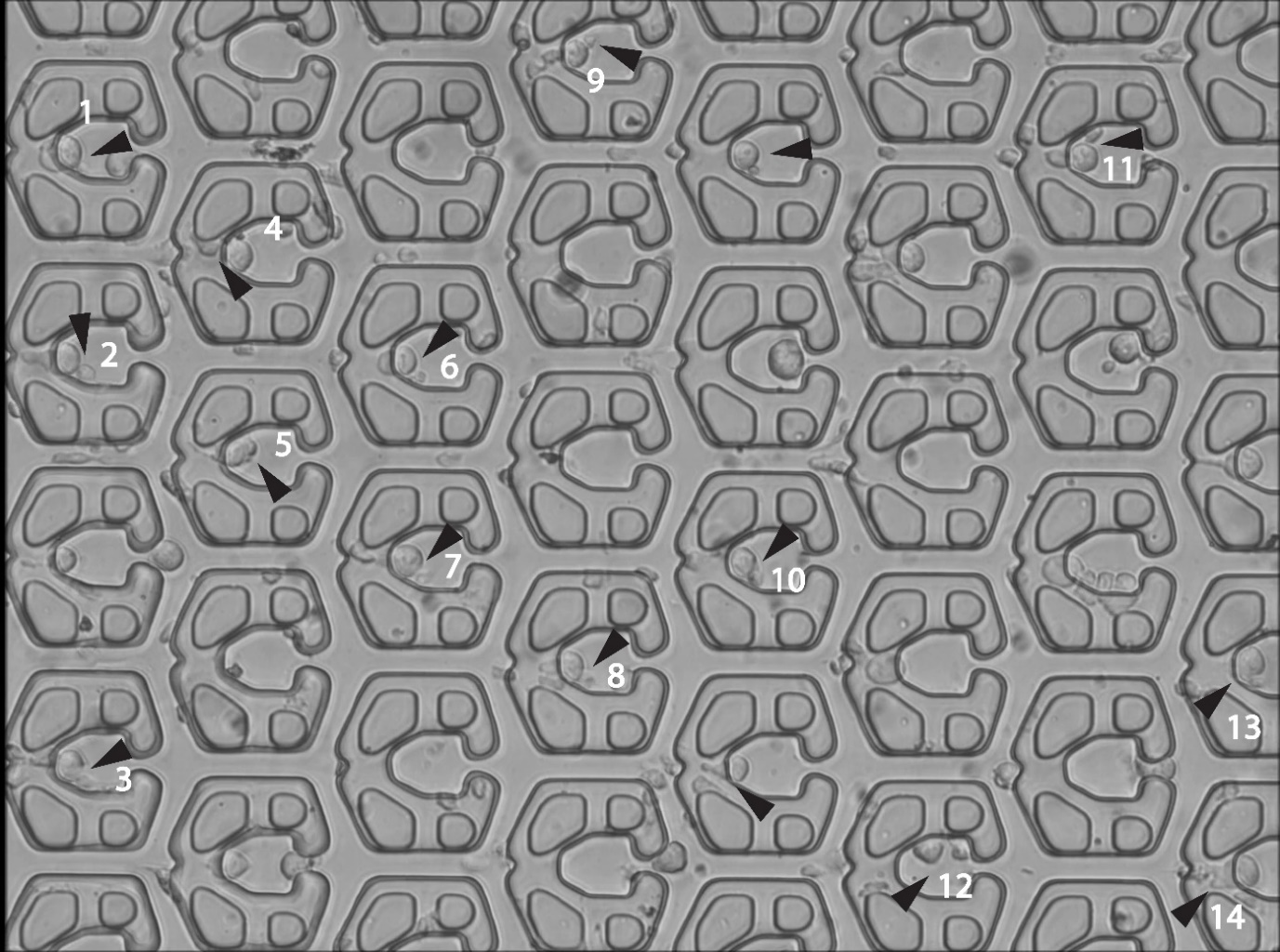


**Supplementary figure S2 (a)**: **Calculation of pairing efficiency with respect to supplementary video 3.**

Total no of traps visible in the frame = 36

Total no of single T cells (in red) which are in contact with macrophages (in green) =14

% Paring efficiency in video frame: (14/36) x 1100= ~ 39%


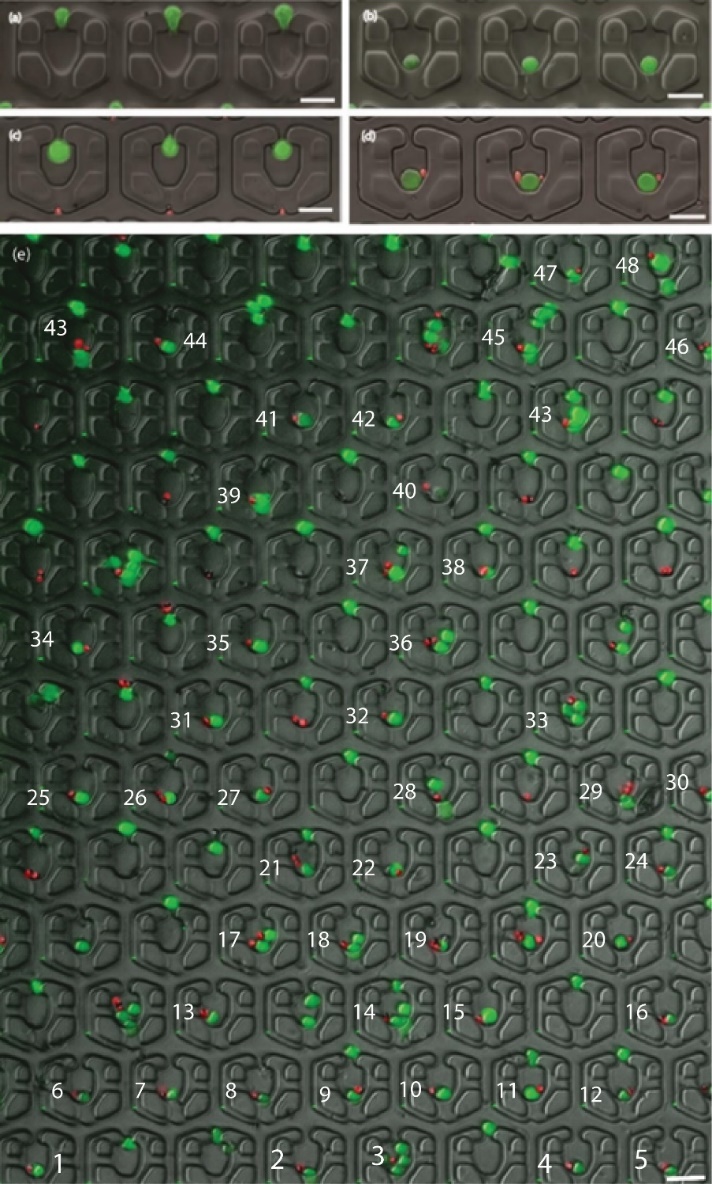


**Supplementary figure S2 (b):** Calculation of pairing efficiency with respect to figure 4

**Calculations of pairing efficiency:**

Total no of traps visible in the frame = 108

Total no of single T cells (in red) which are in contact with macrophages (in green) =45

% Paring efficiency in still frame in lower magnification: (45/108) x 1100=41.66 %

Hence approximately pairing efficiency was concluded to be 40-45%.

| **Serial No** | **Support Pillar heights (Ph )** | **Total channel height (Th)** | **Ratio(X): Ph/Th** |
| --- | --- | --- | --- |
| 1 | 3 micrometer | 15 micrometer | 0.22 |
| 2. | 4 micrometer | 15 micrometer | 0.28 |
| 3. | 5 micrometer | 15 micrometer | 0.33 |

**Supplementary Table T1:** Variation of support pillar height (Ph) with Total channel height (Th) kept constant. X is the ratio of support pillar height (Ph) to Total channel height (Th).

| **Large cell Focusing length (Fl)** | **Small cell Focusing Length (Fs)** | **Ratio (X): Ph/Th** | **Pairing efficiency (%)** |
| --- | --- | --- | --- |
| 24 µm | 20 µm | 0.22 | ~ 33 |
| 24 µm | 20 µm | 0.28 | ~ 40 |
| 28 µm | 20 µm | 0.22 | ~ 27 |
| 28 µm | 20 µm | 0.28 | ~ 30 |

**Supplementary Table T2**: Pairing efficiencies with respect to varying focusing length (FL) and Ratio (X) values.


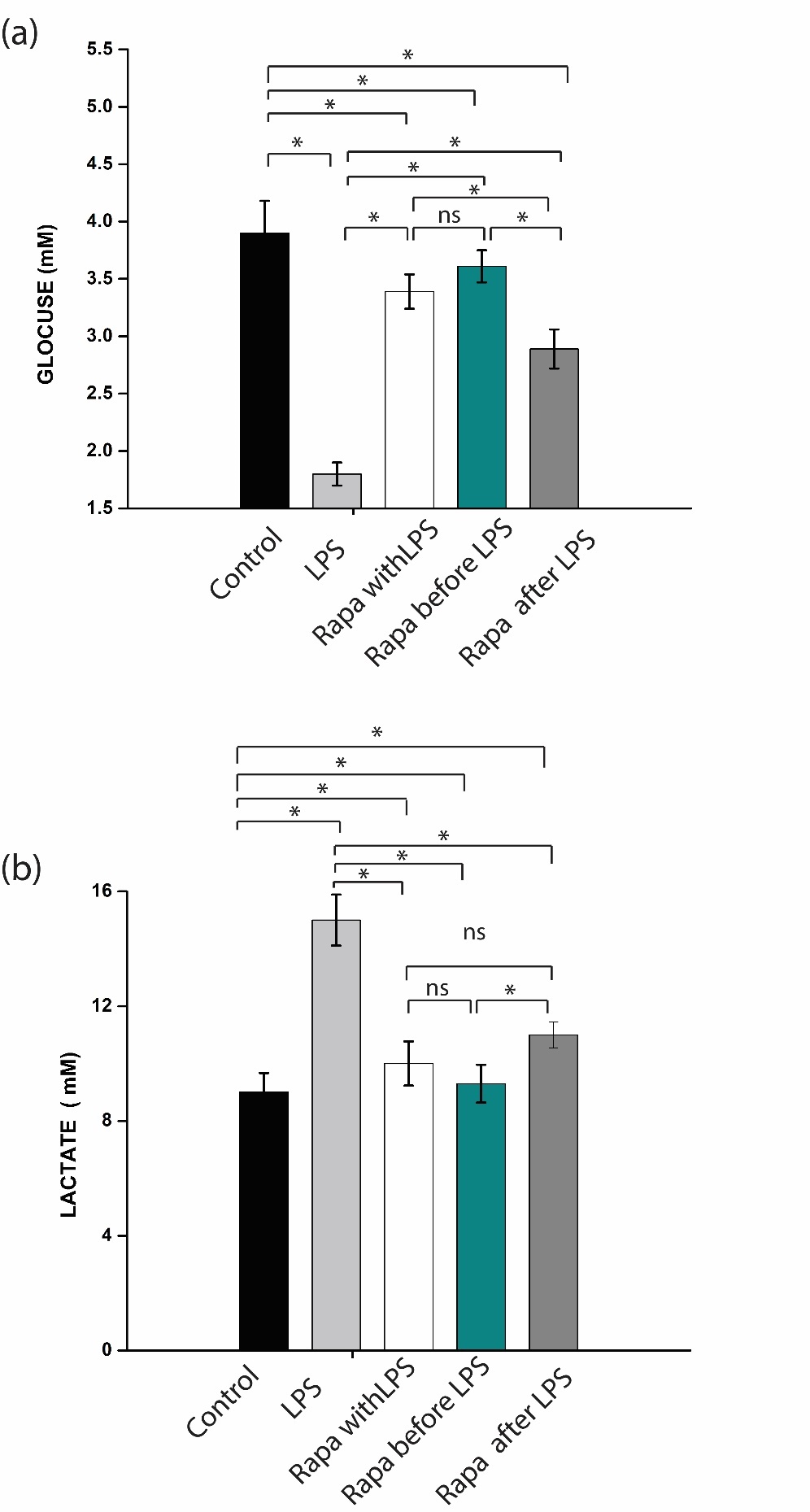


**Figure S3:** **The effect of rapamycin treatment in primary mouse macrophages on glucose metabolism and lactate formation in presence of LPS** (a) Glucose in media after culture (b) Lactate in media after culture, the significant difference is indicated using a ‘*’ and nonsignificant using ’ns’. Significant differences determined with one-way analysis of variance test. *P<0.05.


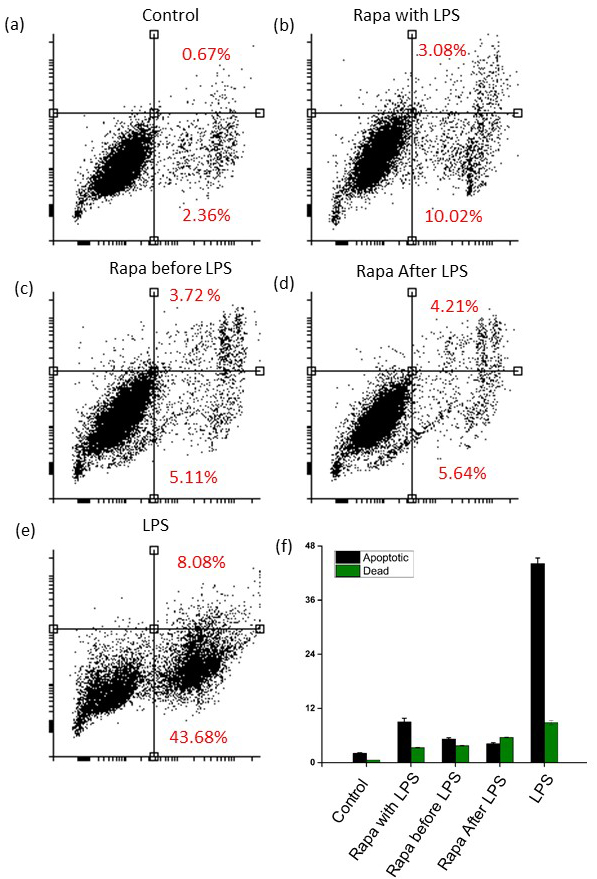


**Figure S4:** **Annexin V FITC- PI Assay after 96 hours of treatment for analysis number of Apoptotic and dead macropahges .**(a) No treatment Control .Macrophages were cultured in media (b) Macropages treated with Rapamycin with LPS (c) Macropages treated with Rapamycin before LPS (d) Macropages treated with Rapamycin after LPS ( e) Macropages treated with LPS (e) Bargraph for comparing apoptotic and dead polulation of macropgaes after treatment .


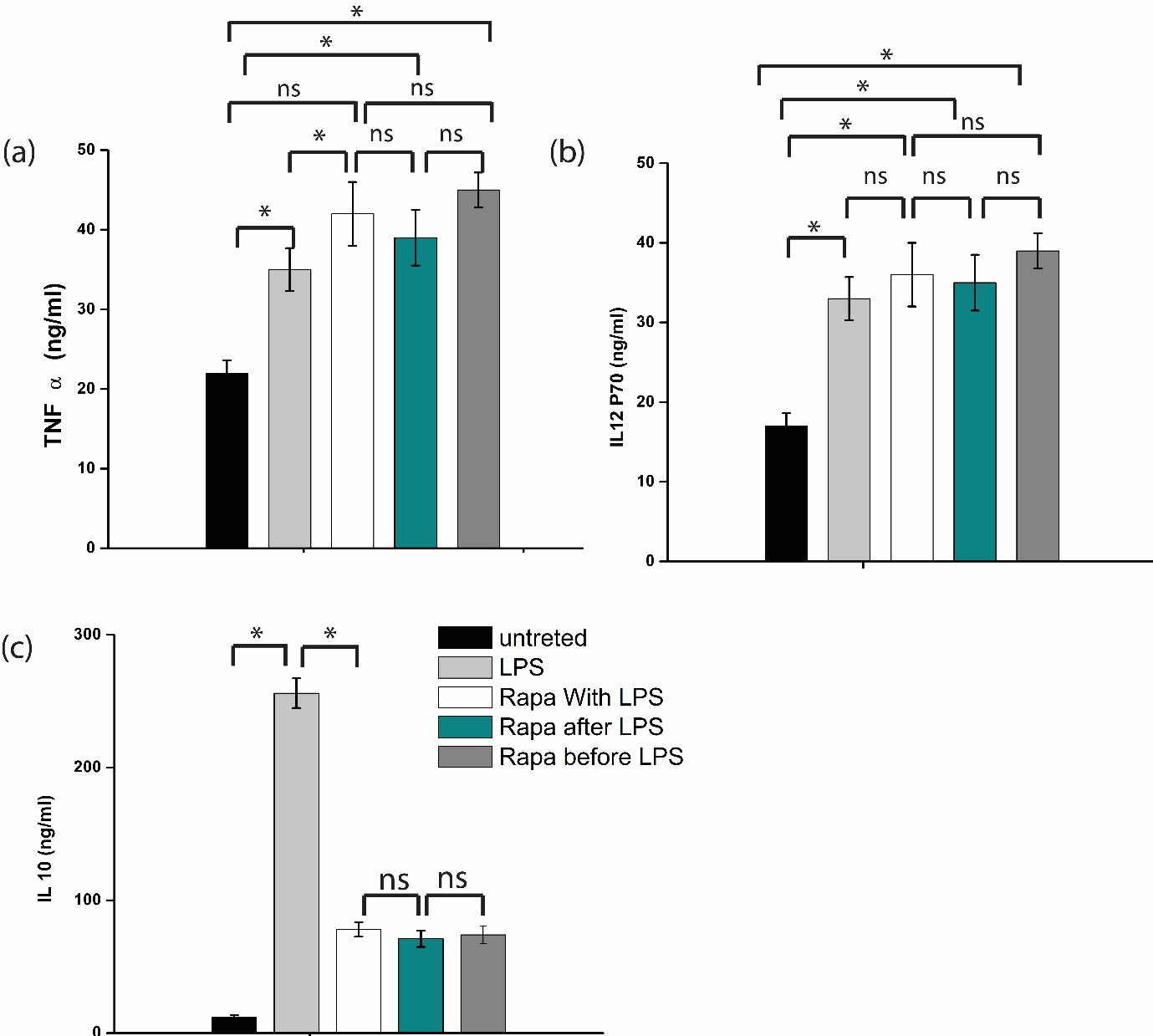


**Figure S5: Pro-inflammatory and anti-inflammatory response by primary mouse macrophages were stimulated with LPS, LPS with rapamycin and LPS after rapamycin for 24 hours (a)** Proinflammatory cytokine TNF α **(b)** Proinflammatory cytokine IL12 p 70 secreted by primary macrophages **(c)** Regulatory cytokine IL10 secreted by primary macrophages. ns= not significant, *= significant. Significant differences determined with one-way analysis of variance test. *P<0.05.


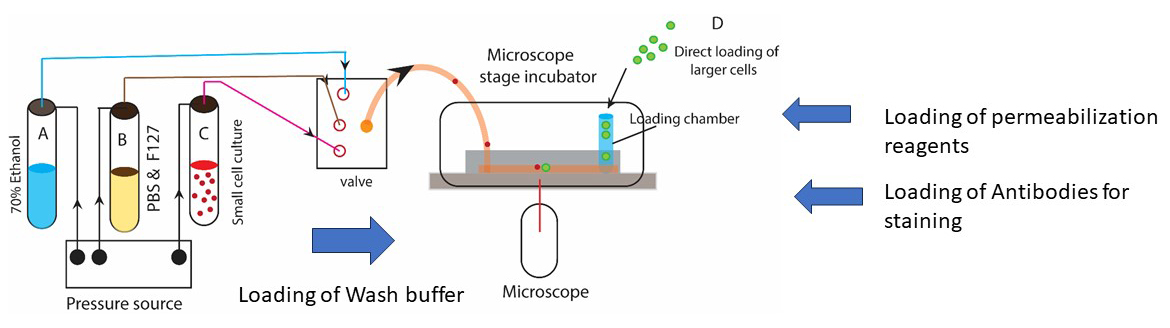


**Figure S6: Schematic of setup of microfluidic chip operations.** The microfluidic device has two ends. On one end, the device is connected to three different fluid reservoirs through multi-channel valves. The reservoirs are connected to a common pressure source which can generate both negative and positive pressures for flowing in fluid and withdrawing fluid from the other side of the microfluidic device. Three chambers containing ethanol, F127 and cells are pumped into chip using microfluidic valving setup. Ethanol is first pumped into chip using positive pressure to remove bubbles and the related valve line is opened (blue line). This is followed by pumping 5% f127 dissolved in PBS (grey line). The other end is kept free for loading cells and withdrawing different simulation agents into the device. First larger cells (macrophages) are pumped into chip using a negative pressure generated by opening the grey line valve used for pumping in F127 into chip. Smaller cells (T cells) are them pumped into the chip using positive pressure for pairing with macrophage cells from the other end. The permeabilization experiments were carried out by withdrawing permeabilization reagent into the device followed by dispensing of washing buffer into the microfluidic device. The antibodies were then withdrawn into the device followed by washing step as shown using arrows. The steps for buffer and reagent exchange are also indicated using arrows.


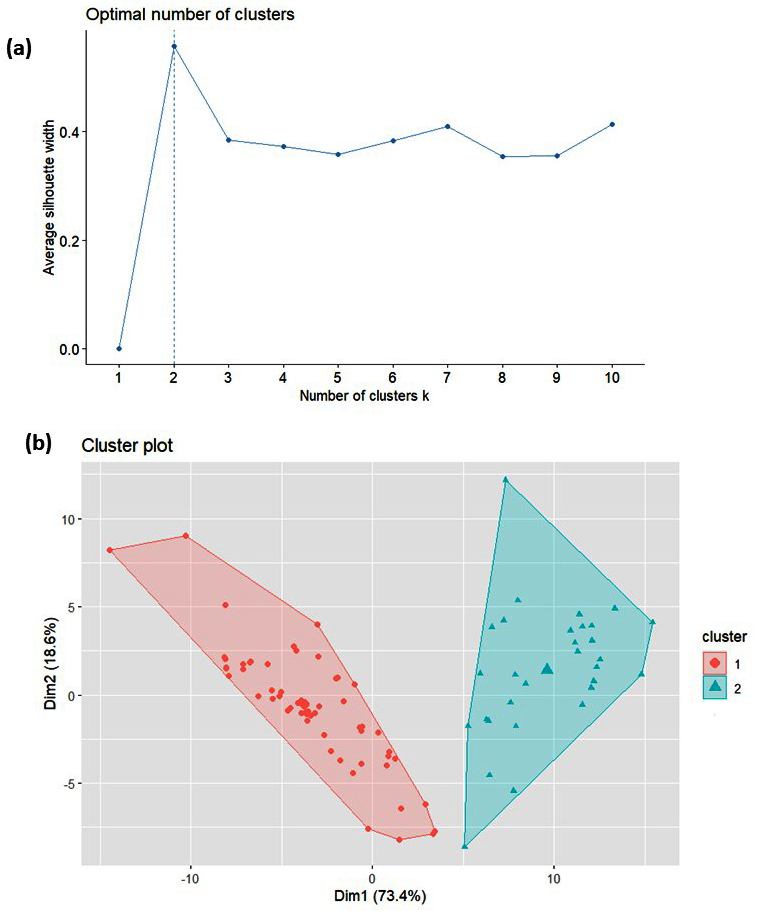


**Supplementary Figure 7:** Determination of average silhouette scores in K means clustering of calcium responses in T cells. (a) Silhouette plot for optimal K value (b) Clustering of strong and weak responders in two distinct groups based on integrated calcium levels.
